# *Ecklonia cava* Ameliorates Cognitive Impairment on Amyloid β-Induced Neurotoxicity by Modulating Oxidative Stress and Synaptic Function in Institute of Cancer Research (ICR) Mice

**DOI:** 10.3390/antiox13080951

**Published:** 2024-08-06

**Authors:** Hyo Lim Lee, Min Ji Go, Han Su Lee, Ho Jin Heo

**Affiliations:** Division of Applied Life Science (BK21), Institute of Agriculture and Life Science, Gyeongsang National University, Jinju 52828, Republic of Korea; gyfla059@gnu.ac.kr (H.L.L.); alswl9245@gnu.ac.kr (M.J.G.); ns3005@gnu.ac.kr (H.S.L.)

**Keywords:** *Ecklonia cava*, marine polyphenol, dieckol, antioxidants, neuroinflammation, cholinergic system, Alzheimer’s disease

## Abstract

This study investigated the neuroprotective effect of 70% ethanol extract of *Ecklonia cava* (EE) in amyloid beta (Aβ)-induced cognitive deficit mice. As a result of analyzing the bioactive compounds in EE, nine compounds were identified using ultra-performance liquid chromatography–quadrupole time-of-flight mass spectrometry (UPLC-Q-TOF-MS). In particular, the diekcol content was quantified by high-performance liquid chromatography with diode-array detection (DAD-HPLC). Biochemical analysis was performed on brain tissue to determine the mechanism of the cognitive function improvement effect of EE. The result showed that EE ameliorated learning and memory decline in behavioral tests on Aβ-induced mice. EE also attenuated oxidative stress by regulating malondialdehyde (MDA) content, reduced glutathione (GSH), and superoxide dismutase (SOD) levels. Similarly, EE also improved mitochondrial dysfunction as mitochondrial membrane potential, ATP production, and reactive oxygen species (ROS) levels. In addition, EE enhanced synapse function by modulating acetylcholine-related enzymes and synaptic structural proteins in the whole brain, hippocampus, and cerebral cortex tissues. Also, EE regulated Aβ-induced apoptosis and inflammation through the c-Jun N-terminal kinase (JNK) and nuclear factor-kappa B (NF-κB) signaling pathways. Furthermore, EE protected neurotoxicity by increasing brain-derived neurotrophic factor (BDNF) production. These results suggest that EE may be used as a dietary supplement for the prevention and treatment of Alzheimer’s disease (AD).

## 1. Introduction

Recent studies have projected that the social and economic burden of Alzheimer’s disease (AD) and related dementias will increase dramatically in the coming decades [1]. AD is characterized by a spectrum of symptoms including cognitive decline, such as memory loss, deterioration of visual abilities, impaired performance, and alterations in behavior [2]. AD is observed not only with behavioral disorders but also profound pathological changes in the brain, such as amyloid plaque accumulation, tau hyperphosphorylation, neuroinflammation, neuronal loss, and neurotransmission disorders [3]. The amyloid beta (Aβ) peptide, which is the major cause of AD, is formed when the amyloid precursor protein (APP) is processed via the β-secretase pathway to produce Aβ_1–40_ or Aβ_1–42_ forms [4]. Oxidative stress, a major cause of AD progression, can be aggravated by metal ions binding to the hydrophilic N-terminus of Aβ peptides and generating a large amount of reactive oxygen species (ROS) [5]. Excessive oxidative stress causes mitochondrial dysfunction, generates more ROS, and reduces mitochondrial membrane potential (MMP), thereby reducing ATP production efficiency [6]. As a result, apoptosis is mediated, resulting in brain cell loss, which is a major feature of degenerative brain diseases such as AD. In addition, ROS by Aβ causes neuroinflammation, which contributes to increasing the permeability of the blood–brain barrier (BBB) [4,5]. Previous studies have reported that Aβ can directly affect vascular endothelial cells and induce destruction of the BBB, and thus cause cognitive dysfunction [7]. In addition, Aβ itself can damage cholinergic neurons by changing the structure and function of synapses [8]. As a result, it reduces the neurotransmitter acetylcholine (ACh) concentration and can cause memory and learning disorders [9]. Although much research has been conducted on the Aβ hypothesis as the cause of AD, there is currently no definitive treatment to prevent or cure it.

Medicinal plant materials have been used for hundreds of years to prevent and treat various diseases [5]. Among them, marine polyphenols, which are plant compounds contained in seaweed, are known to have high antioxidant activity [10]. In particular, phlorotannins, including eckol, bieckol, dibenzodioxin-fucodiphloroethol, dieckol, and triphlorethol A, which are polyphenols abundant in brown seaweed, have been reported to have various physiological activities such as antioxidant, anti-inflammatory, anti-allergic, and neuroprotective activities [10,11]. *Ecklonia cava* (*E. cava*) is an edible marine brown alga that mainly lives off the coast of Jeju Island, Ulleungdo, and Dokdo in Korea. It has been reported that *E. cava* is rich in phlorotannins and fucoidan (a water-soluble dietary fiber) and is effective in improving neurodegenerative diseases [12]. In addition, our previous studies have shown that the water extract of *E. cava* improves fine particulate matter (PM_2.5_)-induced cognitive deficits [13], and phlorotannins and fucoidan extracted from *E. cava* prevent Aβ-induced cognitive impairment [14]. Through this, it was confirmed that the protective effect of *E. cava* against neurodegenerative disorders is due to the phlorotannins. Therefore, we extracted *E. cava* with 70% ethanol instead of water to increase the phlorotannin content, thereby securing potential industrial applications such as a functional food. Furthermore, in the present study, we aimed to investigate further how the 70% ethanol extract of *E. cava* plays a modulatory role in cognitive function.

## 2. Materials and Methods

### 2.1. Materials and Reagents

Aβ peptide 1-42 (PP69), 1,1,3,3-tetramethoxypropane, ethylene-diamine-tetraacetic acid (EDTA), o-phthaldialdehyde (OPT), ethylene glycol-bis(β-aminoethyl ether)-N,N,N′,N′-tetraacetic acid (EGTA), hydroxyethyl piperazine ethane sulfonic acid (HEPES) sodium alt, dichloro-dihydro-fluorescein diacetate (DCF-DA), 1,1′,3,3′-tetraethyl-5,5′,6,6′-tetrachloroimidacarbocyanine iodide (JC-1), 5,5′-dithiobis-2-nitrobenzoic acid (Ellman’s reagent or DTNB), and all other chemicals used were purchased from Sigma-Aldrich Chemical Co. (St. Louis, MO, USA). A standard compound of dieckol was purchased from ChemFaces (Wuhan, China).

### 2.2. Preparation of Extract of E. cava

The 70% ethanol extract of *E. cava* (EE) powder used in the experiment was provided by Threebrooks therapeutics Co., Ltd. (Pohang, Republic of Korea), on 13 October 2023. The water extract of *E. cava* (WEE) was prepared as previously described [13] for high-performance liquid chromatography with diode-array detection (DAD-HPLC) analysis.

### 2.3. Physiological Compounds Analysis

#### 2.3.1. Ultra-Performance Liquid Chromatography–Quadrupole Time-of-Flight Mass Spectrometry (UPLC-Q-TOF-MS) System

EE was dissolved in 50% methanol, filtered by a 0.45 mm syringe filter (Sartorius, Göttingen, Germany), and analyzed using a UPLC-Q-TOF-MS^E^ system (Vion, Waters Corp., Milford, MA, USA) with a BEH C_18_ column (Waters Corp.). The flow rate was set to 0.35 mL/min, and the oven temperature was set to 40 °C in the negative mode. Solvent gradient and MS conditions were set as previously described [13].

#### 2.3.2. DAD-HPLC System

HPLC analysis was performed to quantitatively analyze the content of dieckol in EE and water extract of *E. cava* (WEE). EE and WEE were dissolved in 50% methanol, filtered by a 0.45 mm syringe filter (Sartorius, Göttingen, Germany), and analyzed using a DAD-HPLC system (Ultramate 3000 series, Dionex, Sunnyvale, CA, USA) with a YMC-Triart C_18_ column (150 × 4.6 mm, 5 µm particle size, YMC, Seongnam, Republic of Korea) at 35 °C. The solvent gradient was set to 75% of distilled water containing used 0.1% formic acid and 25% of acetonitrile containing 0.1% formic acid at a flow rate of 1 mL/min for 40 min. The UV spectra were recorded from 210 to 310 nm and exhibited maximum wavelengths at 254 nm. EE and WEE were compared with the dieckol standard compound based on their retention times (RTs) and UV spectra, and quantified utilizing a calibration curve.

### 2.4. Animals and Treatment

All mouse husbandry procedures and operations in this study were approved by the Institutional Animal Care and Use Committee (IACUC) of Gyeongsang National University guidelines (Certificate No. GNU-231005-M0192) on 5 October 2023. ICR male mice (4 weeks old) were purchased from Samtako (Osan, Republic of Korea). The mice were kept in environments with 12/12 h light/dark cycles at 22 ± 2 °C and unlimited access to food and water. The mice were randomly assigned to each stage and allowed to acclimate for one week. They were then divided into five groups of 20 mice each and as follows: normal control (NC), Aβ, EE25, EE50, and EE100 groups. The NC and Aβ groups were orally administered drinking water, and the EE25, EE50, and EE100 groups were orally administered EE 25 mg/kg of body weight (BW), 50 mg/kg of BW, and 100 mg/kg of BW, respectively, for 3 weeks. Afterward, the mice were injected intracerebroventricularly (i.c.v.) with 10 μL of 410 pM Aβ (dissolved in 0.89% NaCl) using a Hamilton microsyringe fitted with a 26-gauge needle to a depth of 2.5 mm at the bregma except for the NC group [14]. Instead, the NC group was injected 10 μL of 0.89% NaCl in the same way. After Aβ injection, a 3-day recovery period was followed by behavioral tests.

### 2.5. Behavioral Tests

#### 2.5.1. Y-Maze

The short-term or working memory was evaluated by recording spontaneous alternation behavior in the Y-maze test, as previously described. The Y-maze consists of 3 arms made of black plastic aligned at equal angles to form a ‘Y’ shape. Mice were placed on one of the arms and allowed to move freely for 8 min, which was recorded using a video tracking system (SMART 3.0, Panlab, Barcelona, Spain). The alternation behavior force was calculated by the following equation:

Alternation behavior (%) = [(number of alternations)/(total number of arm entries − 2)] × 100 (1)

#### 2.5.2. Passive Avoidance (PA)

The short-term or long-term memory based on negative reinforcement was assessed using the PA test, as previously described. The passive avoidance apparatus is made of a lighted and darkened chamber, separated by a central manual door. On the first day of the training trial, mice were acclimated to the dark for 1 min and placed in bright light for 2 min. The time it took for them to open the central door and enter the dark chamber was measured, and an electric shock was immediately administered. When the central divider door was opened, and the mice entered the dark chamber, an electric foot shock (0.5 mA) was delivered for 3 s. The next day of the test trial, under the same conditions, the delay time until the rat re-entered the dark chamber was recorded for up to 5 min.

#### 2.5.3. Morris Water Maze (MWM)

The long-term or spatial memory was assessed using the MWM test, as previously described. The MWM pools a stainless-steel circular pool (diameter: 90 mm, height: 450 mm) divided into four zones of equal area (E, W, S, and N). The pool was filled with squid ink dissolved in water and a black platform was placed in the S zone under the water. On the first day of the experiment, the platform was placed visible on the surface of the water, and the mice were made to swim for 1 min to learn the location of the platform. The next day, the pool was filled with water to obscure the platform from view. Then, mice were placed at the farthest point from the platform, and the time it took to reach the platform was measured for up to 1 min. If the mice were unable to locate the platform, they were gently guided to the platform and placed on it for 20 s to learn. After 4 days of training, in the probe test, the platform was removed, measuring the time it took to stay in the S zone for 90 s. All mice movements were recorded using a video tracking system (SMART 3.0, Panlab, Barcelona, Spain).

### 2.6. Antioxidant Systems in Brain

#### 2.6.1. Malondialdehyde (MDA) Content

MDA content was determined to detect lipid peroxidation in the brain by thiobarbituric acid reactive substance (TBARS) assay. Brain tissues were mixed with PBS equivalent to 10 times the tissue weight and homogenized with beads. The brain homogenates were centrifuged at 2500× *g* for 10 min. Then, 1% phosphoric acid and 0.67% thiobarbituric acid (TBA) were added to the supernatant and boiled at 95 °C in a water bath. After 1 h, the reactants were cooled and measured for absorbance at a wavelength of 532 nm. MDA content was calculated using a standard curve of 1,1,3,3-tetramethoxypropane.

#### 2.6.2. Reduced Glutathione (GSH) Level

Reduced GSH level was measured to evaluate endogenous antioxidants in the brain. Brain tissues were mixed with 10 mM phosphate buffer containing 1 mM EDTA (pH 6.0) equivalent to 10 times the tissue weight and homogenized with beads. The brain homogenates were centrifuged for 15 min at 10,000× *g*. Then, the supernatants were mixed with 1 mg/mL of OPT, 0.26 M Tris-HCl (pH 7.6), and 0.65 N NaOH and reacted at room temperature for 15 min. The reactants were measured for fluorescence at an excitation wavelength of 320 nm and an emission wavelength of 420 nm. Reduced GSH levels were calculated compared to the fluorescence intensity of the NC group (100%).

#### 2.6.3. Superoxide Dismutase (SOD) Level

SOD level was performed to assess endogenous antioxidants in the brain by water-soluble tetrazolium (WST)-1 assay. Brain tissues were mixed with PBS equivalent to 10 times the tissue weight and homogenized with beads. The brain homogenates were centrifuged for 10 min at 10,000× *g*. Afterward, the supernatants were discarded, and the pellet was mixed with a 1× cell extraction buffer for 30 min on ice. The mixtures were centrifuged at 400× *g* for 10 min. The SOD level of the supernatants was detected with a commercial SOD kit (Dojindo Molecular Tech., Rockville, MD, USA).

### 2.7. Mtitochondrial Function in Brain

#### 2.7.1. Mitochondrial ROS Level

Mitochondrial ROS level was measured to assess mitochondrial oxidative damage in the brain. The isolation of mitochondria from brain tissues was performed as previously described. The mitochondria extracts from brain tissues were mixed with a respiration buffer (125 mM KCl, 20 mM HEPES sodium salt, 2 mM KH_2_PO_4_, 1 mM MgCl_2_, 500 μM EGTA, 2.5 mM malate, and 5 mM pyruvate). Then, the mixture was reacted with 25 μM DCF-DA for 20 min. The reactants were measured for fluorescence at an excitation wavelength of 485 nm and an emission wavelength of 530 nm.

#### 2.7.2. MMP

MMP was detected to determine membrane permeabilization and apoptosis due to oxidative damage in the brain. The mitochondria extracts from brain tissues were incubated with a mitochondrial isolation buffer containing 1 mM JC-1 at room temperature for 20 min. Then, the reactants were detected for fluorescence at an excitation wavelength of 530 nm and an emission wavelength of 590 nm.

#### 2.7.3. Mitochondrial ATP Content

Mitochondrial ATP content was measured to evaluate the mitochondrial function of the brain using an ATP bioluminescence assay kit (Promega, Madison, WI, USA). ATP was detected by measuring the light produced by luciferase oxidized by ATP using a luminometer (Promega). ATP content was calculated using a standard curve.

### 2.8. ACh Content and Acetylcolinesterase (AChE) Activity

ACh content was measured to evaluate the cholinergic system in the brain according to the method of Vincent. Brain tissues were mixed with PBS equivalent to 10 times the tissue weight and homogenized with beads. The brain homogenates were centrifuged at 14,000× *g* for 30 min. The supernatants were mixed with alkaline hydroxylamine reagent for 1 min. The alkaline hydroxylamine reagent was prepared by mixing 2 M hydroxylamine in HCl and 3.5 NaOH at room temperature for 3 h. Afterward, the reactants were mixed with 0.5 N HCl and 0.37 M FeCl_3_·6H_2_O (dissolved in 0.1 N HCl) and measured for absorbance at a wavelength of 540 nm.

AChE activity was performed to evaluate the cholinergic system in the brain according to the method of Ellman. The brain supernatant was incubated with a 50 mM sodium phosphate buffer (pH 8.0) at 37 °C. After 15 min, the reactants were mixed with a substrate solution (0.5 mM acetylthiochonine and 1 mM DTNB) and detected for absorbance at a wavelength of 405 nm.

### 2.9. Western Blot Analysis

The experimental process for Western blotting was performed as previously described. Briefly, brain tissues were homogenated in a ProtinEx Animal cell/tissue extraction buffer (Gene All Biotechnology, Seoul, Republic of Korea) containing 1% protease inhibitor cocktail (Thermo Fisher Scientific, Waltham, MA, USA). Then, the homogenates were centrifuged at 13,000× *g* for 10 min, and the supernatant was quantified by a Bradford assay (Bio-Rad, Hercules, CA, USA). Equal amounts of protein samples were separated onto the SDS polyactylamide gel, transferred to a PVDF membrane (Millipore, Burlington, MD, USA), and blocked with 5% skim milk. The membranes were incubated with primary antibodies (1:1000) overnight at 4 °C. Afterward, the membranes were incubated with secondary antibodies (1:3000) at room temperature for 1 h. The intensity of protein bands was visualized using an enhanced chemiluminescence (ECL) detection reagent (Translab, Daejeon, Republic of Korea), and measurements were performed using an iBright CL1000 imager (Thermo Fisher Scientific). The band density of each specific protein was normalized to that of β-actin. Antibody details are shown in Table 1.

### 2.10. Statistical Analysis

The data were expressed as mean ± SD. The statistical analyses performed were one-way analysis of variance (ANOVA) and Duncan’s multiple range test using the SAS program (Ver. 9.4 SAS Institute, Cary, NC, USA). The statistical difference (*p* < 0.05) of each group is indicated by different lowercase letters in the bar or line graphs.

## 3. Results

### 3.1. Physiological Compound of EE

**Figure 1:**
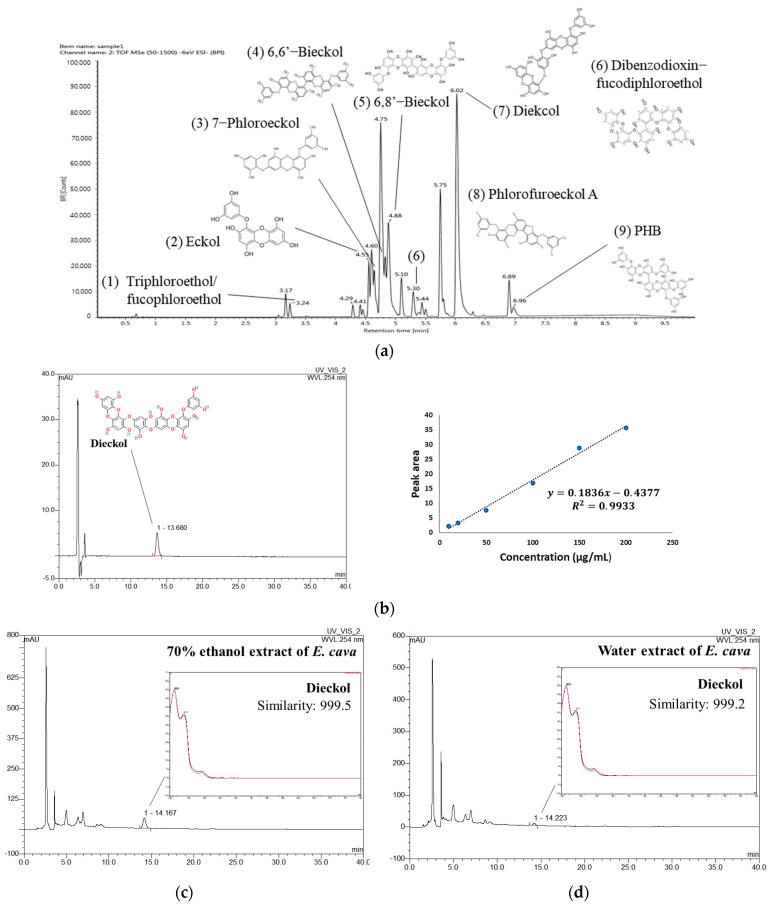
UPLC-Q–TOF/MS^E^ chromatogram of 70% ethanol extract of *Ecklonia cava* (EE) (**a**). HPLC chromatogram of dieckol standard compound with calibration curve (**b**) and HPLC chromatogram of EE (**c**) and water extract of *Ecklonia cava* (WEE) (**d**) at 254 nm.

UPLC-Q-TOF-MS in negative ion mode detected identifiable chromatographic peaks (Figure 1a), including nine phenolic compounds in EE. The proposed compounds were identified by comparison with UPLC-MS or MS^2^ fragments of previous studies [13,15,16,17,18] and the PubChem database. Triphloroethol/fucophloroethol, Eckol, 7-phloroeckol, 6,6′-Bieckol, 6,8′-Bieckol, dibenzodioxin-fucodiphloroethol, dieckol, Phlorofucofuroeckol A, and 2,7″-Phloroglucinol-6,6′-bieckol (PHB) were identified as the main phenolic compounds in EE (Table 2).

To compare the content of dieckol in EE and WEE, quantitative analysis was conducted using a DAD-HPLC analysis. As a result, a dieckol standard compound was identified at 13.680 min, and the coefficient of determination (R^2^) of the calibration curve was 0.9933 (Figure 1b). The peak at 14.167 min in the chromatogram of EE showed a similarity of 0.999 with dieckol (Figure 1c). WEE also showed a similarity of 0.999 with dieckol in the peak that appeared at 14.223 min in the chromatogram (Figure 1d). The result of the quantitative analysis showed that the content of dieckol in EE was 90.89 ± 0.20 μg/mg of dried weight (Table 3). In addition, the content of dieckol in WEE was detected to be 14.08 ± 0.35 μg/mg of dried weight, which was lower than that of EE.

### 3.2. Effect of EE on Behavioral Tests

**Figure 2:**
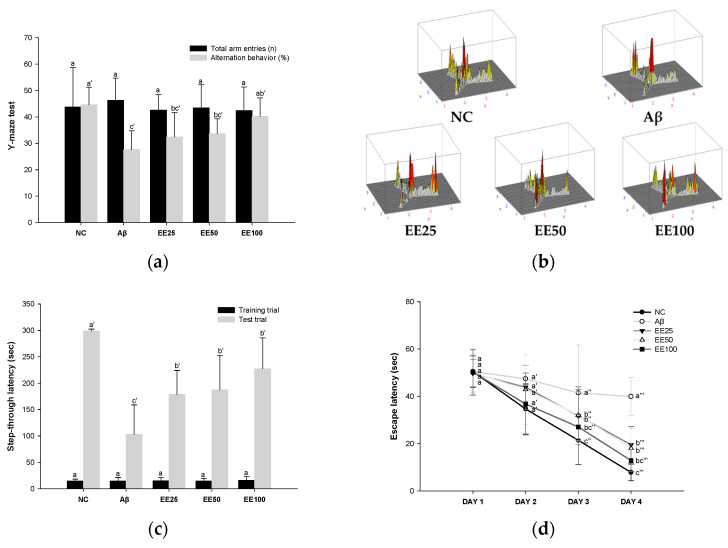

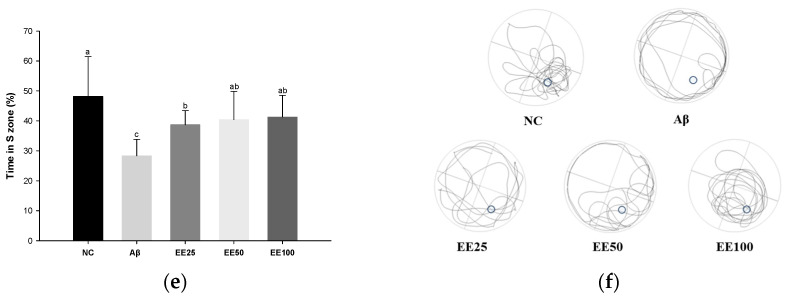
Effect of 70% ethanol extract of *Ecklonia cava* (EE) on behavioral tests. Total arm entries and alternation behavior (%) (**a**) and 3D image of path tracking of groups (**b**) in Y-maze test. Step-through latency (sec) during training and test trials in passive avoidance (PA) test (**c**). Escape latency (sec) during platform trials (**d**), time in S zone on probe test (%) (**e**), and swimming pattern visualization image on probe test (**f**) in Morris water maze (MWM) test. Data are represented as mean ± SD (*n* = 10). Different lowercase letters on the bar or line graph indicate significant differences between groups in each experiment (*p* < 0.05).

In the Y-maze test, there was no significant difference between all groups in the total number of times the mice entered each of the three arms (Figure 2a,b). However, the Aβ group (27.64%) decreased compared to the NC group (44.60%) in the alternation behavior calculated by Equation (1). In contrast, mice fed the EE diet showed increased (EE 25: 32.38%, EE 50: 33.65%, and EE 100: 40.21%) alternation behavior compared to the Aβ group. These results suggest that EE recovered Aβ-induced short-term memory and working memory impairment.

In the PA test, there was no significant difference between all groups in the step-through latency to move from the light chamber to the dark chamber in the training trials (Figure 2c). However, in the test trial, the step-through latency of the Aβ group (103.00 s) was decreased compared to the NC group (298.57 s). In contrast, mice fed the EE diet showed increased (EE 25: 178.71 s EE 50: 187.14 s, and EE 100: 227.29 s) step-through latency compared to the Aβ group. These results suggest that EE recovered Aβ-induced short-term or long-term memory impairment.

In the MWM test, there was no significant difference between all groups in the time taken to find the platform (escape latency) on day 1 (Figure 2d). However, after four days of training, the Aβ group (39.92 s) showed an increased escape latency than the NC group (7.83 s). In contrast, mice fed the EE diet (EE 25: 19.52 s, EE 50: 18.03 s, and EE 100: 12.83 s) had a decreased escape latency compared to the Aβ group. Moreover, in the probe test, the Aβ group (28.33%) stayed in the S zone (%) for a shorter time than the NC group (48.14%) (Figure 2e). However, the EE groups (EE 25: 38.69%, EE 50: 40.41%, and EE 100: 41.24%) had an increased time spent in the S zone compared to the Aβ group. Similarly, the video tracking of the mice showed that the EE groups spent more time in the S zone and a higher number of S zone area crossings than the Aβ group (Figure 2f). These results suggest that EE recovered Aβ-induced long-term memory and spatial memory impairment.

### 3.3. Effect of EE on Antioxidant Systems in Brain

**Figure 3:**
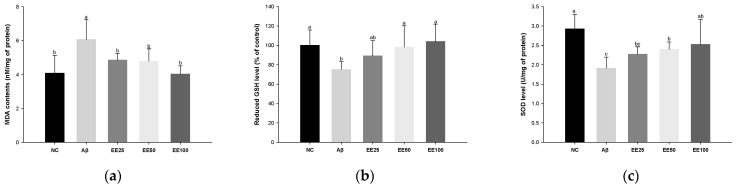
Effect of 70% ethanol extract of Ecklonia cava (EE) on antioxidant system in brain tissues on Aβ-induced mice. Malondialdehyde (MDA) content (**a**), reduced glutathione (GSH) level (**b**), and superoxide dismutase (SOD) level (**c**). Data were represented as mean ± SD (*n* = 7). Different lowercase letters on the bar graph indicate significant differences between groups (*p* < 0.05).

MDA content increased in the Aβ group (6.06 nM/mg of protein) compared with the NC group (4.08 nM/mg of protein) (Figure 3a). However, the MDA content decreased in the EE groups (EE 25: 4.86 nM/mg of protein, EE 50: 4.79 nM/mg of protein, and EE 100: 4.03 nM/mg of protein) compared with the Aβ group.

Reduced GSH level decreased in the Aβ group (1.91 U/mg of protein) compared with the NC group (2.93 U/mg of protein) (Figure 3b). However, the reduced GSH level increased in the EE groups (EE 25: 89.07%, EE 50: 98.24%, and EE 100: 104.09%) compared with the Aβ group.

SOD level decreased in the Aβ group (75.23%) compared with the NC group (100.00%) (Figure 3b). However, the SOD level increased in the EE groups (EE 25: 2.27 U/mg of protein, EE 2.40 U/mg of protein, and EE 100: 2.53 U/mg of protein) compared with the Aβ group.

### 3.4. Effect of EE on Mitochondrial Function in Brain

**Figure 4:**
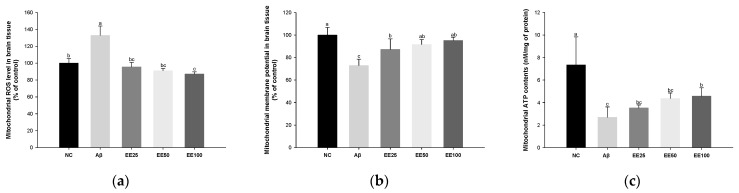
Effect of 70% ethanol extract of Ecklonia cava (EE) on antioxidant system in brain tissues on Aβ-induced mice. Malondialdehyde (MDA) content (**a**), reduced glutathione (GSH) level (**b**), and superoxide dismutase (SOD) level (**c**). Data are represented as mean ± SD (*n* = 5). Different lowercase letters in the bar graph indicate significant differences between groups (*p* < 0.05).

Mitochondrial ROS level increased in the Aβ group (132.87%) compared with the NC group (100.00%) (Figure 4a). However, the mitochondrial ROS level content decreased in the EE groups (EE 25: 95.47%, EE 50: 91.03%, and EE 100: 87.03%) compared with the Aβ group.

MMP reduced in the Aβ group (72.86%) compared with the NC group (100.00%) (Figure 4b). However, MMP increased in the EE groups (EE 25: 87.21%, EE 50: 91.55%, and EE 100: 95.12%) compared with the Aβ group.

Mitochondrial ATP content decreased in the Aβ group (2.69 nM/mg of protein) compared with the NC group (7.34 nM/mg of protein) (Figure 4c). However, the mitochondrial ATP content increased in the EE groups (EE 25: 3.51 nM/mg of protein, EE 50: 4.36 nM/mg of protein, and EE 100: 4.56 nM/mg of protein) compared with the Aβ group.

### 3.5. Effect of EE on Neurotoxicity

**Figure 5:**
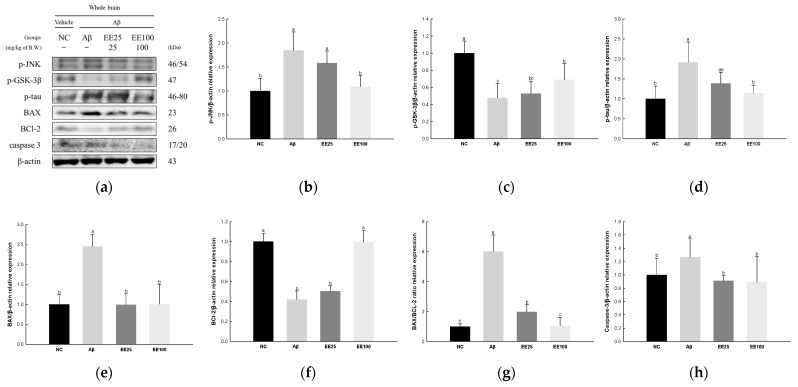
Effect of 70% ethanol extract of Ecklonia cava (EE) on neurotoxicity in Aβ-induced mice. Western blot band images (**a**). Relative expressions of p-JNK (**b**), p-GSK-3β (**c**), p-tau (**d**), BAX (**e**), BCL-2 (**f**), BAX/BCL-2 ratio (**g**), and caspase-3 (**h**) on the corresponding quantitation to β-actin. Data are represented as mean ± SD (*n* = 3). Different lowercase letters in the bar graph indicate significant differences between groups (*p* < 0.05).

The expressions of JNK signaling pathway-related proteins were measured to confirm the protective effect of EE on Aβ-induced neurotoxicity. The result showed that the expressions of p-JNK (1.85), p-GSK-3β (0.48), p-tau (1.91), BAX (2.46), BAX/BCL-2 ratio (6.01), and caspase-3 (1.27) increased in the Aβ group compared with the NC group (1.00) (Figure 5). In contrast, the expression of BCL-2 decreased in the Aβ group (0.42) compared with the NC group (1.00). On the other hand, the EE 25 and EE 100 groups improved in the expressions of p-JNK (1.58 and 1.09, respectively), p-GSK-3β (0.53 and 0.69, respectively), p-tau (1.38 and 1.14, respectively), BAX (0.99 and 1.01, respectively), BCL-2 (0.50 and 0.99, respectively), BAX/BCL-2 ratio (1.97 and 1.05, respectively), and caspase-3 (0.91 and 0.89, respectively) compared with the Aβ group.

### 3.6. Effect of EE on Neuroinflammation

**Figure 6:**
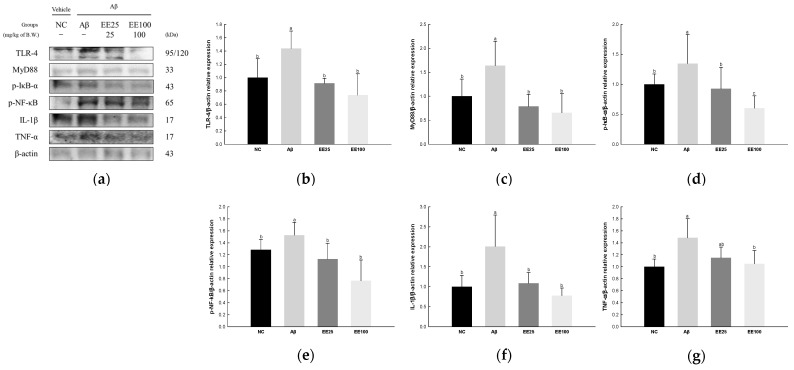
Effect of 70% ethanol extract of Ecklonia cava (EE) on neuroinflammation in Aβ-induced mice. Western blot band images (**a**). Relative expressions of TLR-4 (**b**), MyD88 (**c**), p-IκB-α (**d**), p-NF-κB (**e**), IL-1β (**f**), and TNF-α (**g**) on the corresponding quantitation to β-actin. Data are represented as mean ± SD (*n* = 3). Different lowercase letters in the bar graph indicate significant differences between groups (*p* < 0.05).

The expressions of NF-κB signaling pathway-related proteins were detected to confirm the protective effect of EE on Aβ-induced neuroinflammation. As a result, the expressions of TLR-4 (1.44), MyD88 (1.64), p-IκB-α (1.35), p-NF-κB (1.53), IL-1β (2.01), and TNF-α (1.48) increased in the Aβ group compared with the NC group (1.00) (Figure 6). However, the EE 25 and EE 100 groups had reduced expressions of TLR-4 (0.91 and 0.74, respectively), MyD88 (0.79 and 0.66, respectively), p-IκB-α (0.93 and 0.60, respectively), p-NF-κB (1.13 and 0.77, respectively), IL-1β (1.09 and 0.78, respectively), and TNF-α (1.15 and 1.05, respectively) compared with the Aβ group.

### 3.7. Effect of EE on BBB Dysfunction

**Figure 7:**
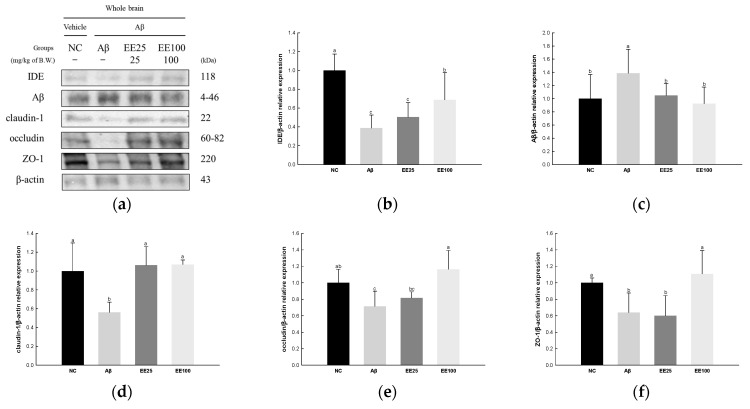
Effect of 70% ethanol extract of Ecklonia cava (EE) on blood–brain barrier (BBB) in Aβ-induced mice. Western blot band images (**a**). Relative expressions of IDE (**b**), Aβ (**c**), claudin-1 (**d**), occludin (**e**), and ZO-1 (**f**) on the corresponding quantitation to β-actin. Data are represented as mean ± SD (*n* = 3). Different lowercase letters in the bar graph indicate significant differences between groups (*p* < 0.05).

The expressions of tight junction proteins were determined to confirm the protective effect of EE on Aβ-induced BBB dysfunction. The result showed that the expression of Aβ (1.39) increased in the Aβ group compared with the NC group (1.00) (Figure 7). In contrast, the expressions of IDE (0.39), claudin-1 (0.56), occludin (0.72), and ZO-1 (0.64) decreased in the Aβ group compared with the NC group (1.00). However, the EE 25 and EE 100 groups regulated the expressions of IDE (0.51 and 0.69, respectively), Aβ (1.05 and 0.93, respectively), claudin-1 (1.06 and 1.07, respectively), occludin (0.82 and 1.16, respectively), and ZO-1 (0.60 and 1.11, respectively) compared with the Aβ group.

### 3.8. Effect of EE on Neuroplasticity

#### 3.8.1. BDNF/AKT/CREB Pathway

**Figure 8:**
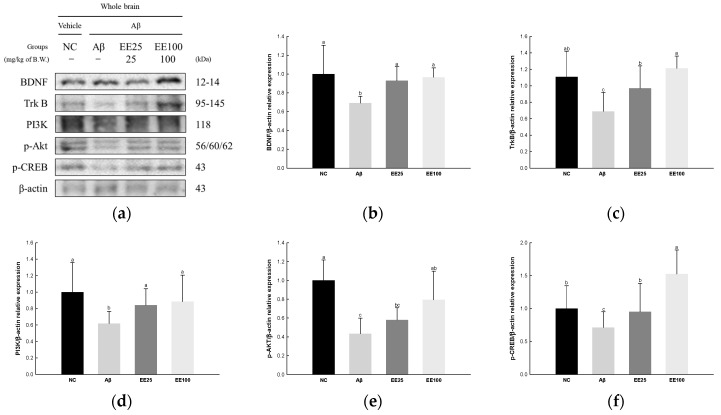
Effect of 70% ethanol extract of Ecklonia cava (EE) on BDNF/AKT/CREB signaling pathway in Aβ-induced mice. Western blot band images (**a**). Relative expressions of BDNF (**b**), TrkB (**c**), PI3K (**d**), p-AKT (**e**), and p-CREB (**f**) on the corresponding quantitation to β-actin. Data are represented as mean ± SD (*n* = 3). Different lowercase letters on the bar graph indicate significant differences between groups (*p* < 0.05).

The expressions of BDNF/AKT/CREB signaling pathway-related proteins were measured to confirm the protective effect of EE on Aβ-induced neuroplasticity damage. The result showed that the expressions of BDNF (0.69), TrkB (0.69), PI3K (0.62), p-AKT (0.43), and p-CREB (0.71) decreased in the Aβ group compared with the NC group (1.00) (Figure 8). However, the EE 25 and EE 100 groups had increased expressions of BDNF (0.93 and 0.96, respectively), TrkB (0.97 and 1.21, respectively), PI3K (0.84 and 0.88, respectively), p-AKT (0.58 and 0.79, respectively), and p-CREB (0.95 and 1.52, respectively) compared with the Aβ group.

#### 3.8.2. Cholinergic System and Synaptic Proteins

**Figure 9:**
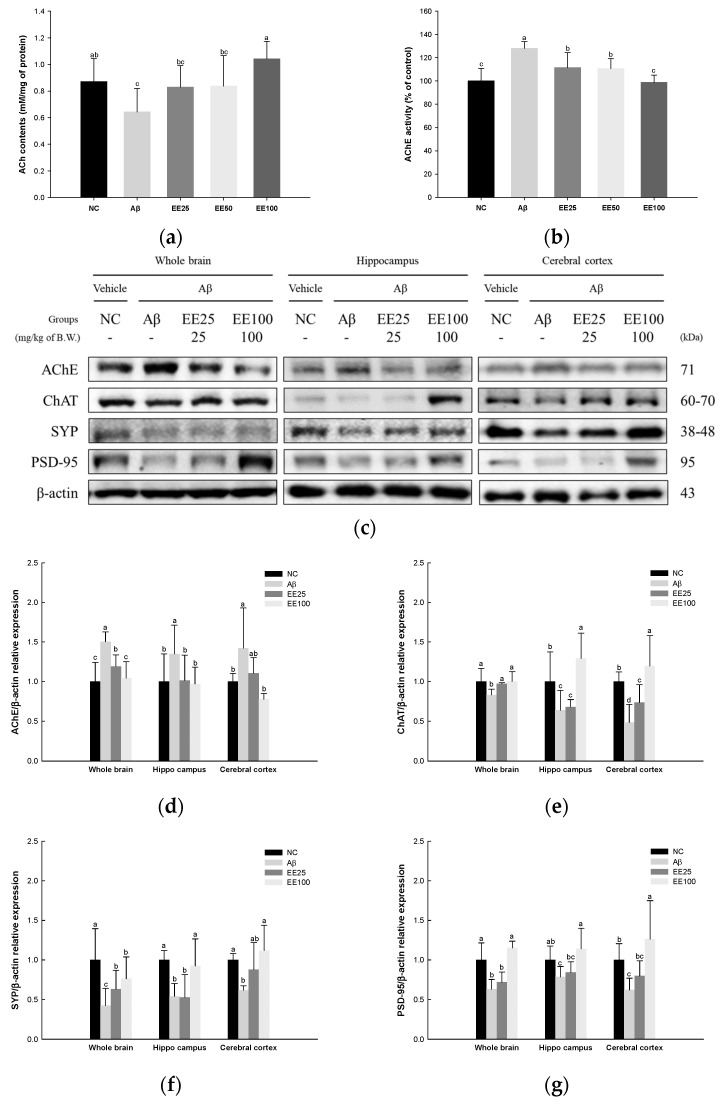
Effect of 70% ethanol extract of Ecklonia cava (EE) on cholinergic system and synaptic proteins in Aβ-induced mice. ACh content (**a**) and AChE activity (**b**) in whole brain tissues, and these data are shown as mean ± SD (*n* = 7). Western blot band images (**c**). Relative expressions of AChE (**d**), ChAT (**e**), SYP (**f**), and PSD-95 (**g**) in whole brain, hippocampus, and cerebral cortex tissues on the corresponding quantitation to β-actin. Data are represented as mean ± SD (*n* = 3). Different lowercase letters in the bar graph indicate significant differences between groups (*p* < 0.05).

ACh content and AChE activity in whole brain tissues and the expressions of cholinergic enzymes and synaptic proteins in whole brain, hippocampus, and cerebral cortex tissues were measured to confirm the protective effect of EE on Aβ-induced neuroplasticity damage. The result showed that the ACh content decreased, while AChE activity increased in the Aβ group (0.64 nM/mg of protein and 128.11%, respectively) compared with the NC group (0.87 nM/mg of protein and 100.00%, respectively) (Figure 9). However, the mice fed EE had increased ACh content (EE 25: 0.83 nM/mg of protein, EE 50: 0.84 nM/mg of protein, and EE 100: 1.04 nM/mg of protein) and decreased AChE activity (EE 25: 111.45%, EE 50: 110.64%, and EE 100: 98.79%).

The results of the Western blot analysis for the expressions of cholinergic enzymes as AChE and ChAT and synaptic proteins as SYP and PSD-95 are presented in Figure 9c–g. The expression of AChE in whole brain, hippocampus, and cerebral cortex tissues increased in the Aβ group (1.50, 1.35, and 1.42, respectively) compared with the NC group (1.00) (Figure 9c,d) while it decreased in the EE 25 (1.19, 1.02, and 1.10, respectively) and EE 100 groups (1.04, 0.97, and 0.78, respectively) compared with the Aβ group. The expression of ChAT in the whole brain, hippocampus, and cerebral cortex tissues decreased in the Aβ group (0.83, 0.64, and 0.48, respectively) compared with the NC group (1.00) (Figure 9c,e) while it was increased in the EE 25 (0.97, 0.68, and 0.74, respectively) and EE 100 groups (1.00, 1.29, and 1.20, respectively) compared with the Aβ group. The expression of SYP in whole brain, hippocampus, and cerebral cortex tissues decreased in the Aβ group (0.42, 0.54, 0.62, respectively) compared with the NC group (1.00) (Figure 9c,f) while it increased in the EE 25 (0.63, 0.53, and 0.88, respectively) and EE 100 groups (0.76, 0.93, and 1.12, respectively) compared with the Aβ group. The expression of PSD-95 in whole brain, hippocampus, and cerebral cortex tissues decreased in the Aβ group (0.63, 0.79, and 0.62, respectively) compared with the NC group (1.00) (Figure 9c,g). On the other hand, it increased in the EE 25 (0.72, 0.84, and 0.80, respectively) and EE 100 groups (1.15, 1.14, and 1.26, respectively) compared with the Aβ group.

## 4. Discussion

The generation and accumulation of Aβ play pivotal roles in brain health such as AD pathology. Aβ plaques are generated by a sequential cleavage of APP by β-secretase and γ-secretase, and Aβ_1–42_ is the most toxic of the generated peptide fragments [19]. The deposition of Aβ aggregates reduces memory and learning ability through oxidative stress, neuroinflammation, neuronal apoptosis, BBB destruction, and synaptic damage [1,2]. Dalley and his colleagues reported that Aβ oligomers cause cognitive dysfunction by disrupting synaptic plasticity, particularly in the hippocampus and prefrontal cortex [20]. Synaptic plasticity declines lead to cholinergic signaling abnormalities, and ultimately to cognitive deficits. Therefore, this study investigated the neuroprotective effects of EE in Aβ-induced cognitive impairment mice model. Furthermore, we secured potential industrial applications by increasing the contents of physiological compounds in *E. cava* compared to our prior study [13].

Marine-derived natural products are materials with enormous potential, including new therapeutics. *E. cava* is a representative edible brown alga that contains various bioactivity molecules such as phlorotannins, fucoxanthin, fucoidan, and alginic acid. Because of this, numerous studies have been conducted on its antibacterial, antioxidant, anti-inflammatory, and anti-diabetic activities. Our previous study confirmed that the phlorotannin and fucoidan extracted from *E. cava* or an extract of *E. cava* improved Aβ-induced and PM_2.5_-induced cognitive dysfunction [13,14]. In addition, we confirmed through UPLC/MS analysis that the neuroprotective effect of the water extract of *E. cava* was due to its abundant phenolic compounds such as eckol, 2-phloroeckol, dieckol, and phlorofucofuroeckol [13]. The activity of phlorotannins against neurodegenerative diseases has already been extensively studied [21,22]. Olasehinde et al. reported that 8,8′-bieckol and 6,6′-bieckol exhibited neuroprotective effects against AD by inhibiting APP biosynthesis and improving neuroinflammation [23]. In particular, it has been reported that dieckol improves cognitive function by inhibiting Aβ production and AChE activity [22,24]. In addition, dieckol inhibited excessive microglial activation and apoptosis through the downregulation of the AKT/extracellular signal-regulated kinase (ERK)/nicotinamide adenine dinucleotide phosphate hydrogen (NADPH) oxidase pathway [25], and exhibited neuroprotective effects against glutamate toxicity through the activation of the nuclear factor erythroid 2-related factor 2 (Nrf2)/heme oxygenase (HO)-1 signaling pathway [26]. Therefore, we extracted *E. cava* with 70% ethanol to increase the content of dieckol, which has a neuroprotective effect. As a result, a total of nine polyphenol compounds were identified in EE, most of which were phlorotannins (Figure 1 and Table 2). Moreover, the peak of dieckol was higher in the 70% ethanol extract than in the water extract of *E. cava*, as shown in a previous study [13]. Likewise, dieckol content in EE was quantitatively analyzed, and the result was 90.89 μg/mg, which was higher than that of the water extract of *E. cava* (14.08 μg/mg) (Table 3). Therefore, this study confirmed the effect and mechanism of EE with increased dieckol content on Aβ-induced cognitive impairment.

AD is the most well-known disease of senile dementia, a chronic neurodegenerative disease characterized by behavioral disorders and cognitive decline due to progressive memory loss. Histopathological features of AD include the accumulation of Aβ and tau tangles, deficits in cholinergic neurotransmission, widespread neuronal loss, and synaptic changes in the cerebral cortex and hippocampus. Although the amyloid and cholinergic hypotheses are known as representative causes of AD, the exact cause has not yet been identified [27,28]. While facing numerous controversies, the amyloid hypothesis continues to evolve and still uses a significant biomarker for dementia [29]. Several studies have reported that Aβ i.c.v. injection causes behavioral abnormalities in mice [30,31]. Similarly, in this study, we confirmed that working memory, spatial perception ability, short-term memory, and long-term memory function were reduced in Aβ-induced mice with Y-maze, PA, and MWM tests (Figure 2). In a previous study, a phlorotannin-rich fraction from *Ishige foliacea* was reported to improve cognitive behavioral disorders through the BDNF/CREB pathway in scopolamine-induced mice [32]. Moreover, Lee et al. reported that *E. cava* polyphenols improved brain fatty acid composition and learning and memory impairment in high-fat diet (HFD)-induced mice [33]. In our previous study, we confirmed that consuming polyphenols isolated from *E. cava* improved trimethyltin (TMT)-induced working memory and long-term memory impairment in mice [34]. Furthermore, mice that consumed dieckol isolated from *E. cava* improved spatial memory and working memory compared to the placebo [35]. Likewise, in this study, mice fed EE improved spontaneous alternating behavior, memory for unpleasant stimuli (electric shocks), and spatial cognitive abilities. Consequently, we considered that EE supplementation could help Aβ-induced cognitive deficits and conducted a mechanism study.

It is already well-known that oxidative stress plays a vital role in the onset and progression of AD [5]. In particular, brain tissue is vulnerable to oxidative stress because it is rich in unsaturated fatty acids and has a high oxygen demand but lacks an antioxidant system compared to other tissues [36]. To combat oxidative stress, the body maintains redox balance with antioxidant enzymes such as SOD, catalase, and GSH peroxidase (GPx), and low-molecular-weight antioxidants such as GSH, vitamins, and carotenoids [5]. However, excessive oxidative stress causes DNA mutations and lipid and protein peroxidation and reduces endogenous antioxidant content in the brain [37]. Moreover, oxidative stress can be further aggravated by the binding of metal ions such as Zn^2+^ and Cu^2+^ to the hydrophilic N-terminus of Aβ peptides, generating large amounts of ROS [5]. As a result, oxidized Aβ proteins can accumulate between synapses and cause neurotoxicity [8]. Indeed, increased levels of Aβ_1-42_ peptide have been reported to be associated with increased lipid oxidation products and decreased antioxidants in the hippocampus and cerebral cortex of AD patients [37,38]. In addition, Wojsiat et al. reported that the antioxidant activity of AD brains is reduced compared to age-matched controls, leading to an oxidant/antioxidant imbalance [38]. Similarly, in this study, the antioxidant system was significantly decreased in Aβ_1-42_-induced mice brain tissues (Figure 3). According to Cui et al., dieckol reduced glutamate-stimulated oxidative stress in primary cortical neurons and HT22 cells via the Nrf2/HO-1 pathway [26]. In a previous study, 50% ethanol extract of *E. cava* and dieckol showed a neuroprotective effect against oxidative stress in dihydrochloride and H_2_O_2_-induced PC12 and SH-SY5Y cells [39]. Moreover, in our prior study, polyphenols isolated from *E. cava* restored SOD activity and reduced GSH content in TMT-induced mice brain tissues [34]. Likewise, in this study, EE ameliorated Aβ-induced antioxidant system imbalance by regulating MDA content, and reduced and SOD levels. These results demonstrate that EE containing phlorotannins, a powerful antioxidant, may have helped prevent cognitive decline by reducing Aβ-induced ROS.

Oxidative stress is also associated with mitochondrial dysfunction. Mitochondria are the primary energy producers in cells, and in this process, a large amount of oxygen is used, and ROS is generated [6]. In normal states, they are easily removed by antioxidants, but when ROS accumulates, the removal rate decreases and mitochondria are damaged [2,6]. The accumulation of Aβ not only interferes with the metabolic activity of neurons around mitochondria but also reduces the efficiency of mitochondrial energy production by disrupting electron transport chain complexes [15,38]. Moreover, Aβ_1-42_ localizes to the mitochondria, which can interact with mitochondrial components and exert cellular toxicity [40]. Ashleigh et al. identified Aβ, APP, and APP β-C-terminal fragments in the mitochondria-rich hippocampal tissue fraction of transgenic APP mice [40]. Dysfunctional mitochondria are known to have reduced ATP production and increased ROS production, contributing to the oxidative imbalance observed in AD [5,38]. In summary, Aβ plaque accumulation causes mitochondrial dysfunction characterized by decreased MMP and ATP production and excess ROS production. Indeed, in the current study, mitochondrial dysfunction was observed in Aβ-induced mice brain tissues (Figure 4). In a previous study, phlorofucofuroeckol improved glutamate-induced PC12 cells neurotoxicity, maintained mitochondrial morphology, and increased MMP [41]. In addition, *E. cava* extracts decreased mitochondrial DNA damage and ROS generation in the aortas of spontaneous hypertensive rats (SHRs) [42]. Our prior study demonstrated that a mixture of phlorotannin and fucoidan from *E. cava* promotes mitochondria activity in Aβ-induced mice [14]. Likewise, in the present study, intake of EE significantly improved MMP, ATP products, and ROS level in Aβ-induced mice. These results established that EE, with a powerful antioxidant effect, may have helped prevent cognitive decline by reducing Aβ-induced mitochondrial dysfunction.

It is now accepted that Aβ and tau act as triggers and bullets in the development of AD [43]. Aβ aggregates activate apoptosis through complex and diverse pathways with oxidative stress, mitochondrial dysfunction, and inflammation [3]. Moreover, GSK-3β is an enzyme involved in the formation of highly phosphorylated tau protein in neurofibrillary tangles (NFTs) and is also involved in neuronal loss in neurodegenerative diseases [33]. When tau protein undergoes hyperphosphorylation and accumulates, it disrupts the stability of microtubules in neurons, contributing to neuronal deformation and damage that can lead to memory impairment [43]. The hyperphosphorylated tau protein formed by various kinases, including JNK, AKT, and microtubule-affinity regulating kinase (MARK), can activate caspase-3 activity and induce neuroapoptosis [44]. The activation of JNK decreases the expression of anti-apoptosis protein BCL-2 and increases the expression of the pro-apoptotic protein BAX [31]. BAX increases the permeability of the mitochondrial membrane, releasing cytochrome c from inside the mitochondria into the cytoplasm [34,40]. Afterward, cytochrome c forms an apoptosome with apoptotic protease activating factor (Apaf)-1, which sequentially activates caspase-9, -3, -6, and -7, thereby inducing apoptosis [3]. Indeed, in our previous study, *E. cava* reduced the expression of p-tau and BAX and inhibited the release of cytochrome c in TMT-induced mice brain [34]. In this study, EE modulated the JNK signaling pathway and decreased p-tau protein in Aβ-induced neuroapoptosis (Figure 5). Similarly, a previous study reported that phlorofucofuroeckol protected against cytotoxicity in a caspase-dependent apoptosis manner in glutamate-induced PC12 cells [41]. Moreover, 7-Phloroeckol from *E. cava* decreased apoptosis-related proteins such as p-JNK, BAX, caspase-9, and caspase-3 and increased BCL-2 in alcohol-induced HepG2/CYP2E cells [45]. These results indicate that EE with antioxidant and mitochondrial protective effects may help with cognitive dysfunction by reducing Aβ-induced neuroapoptosis.

Chronic neuroinflammation increases the risk of developing AD [2]. Neuroinflammation is characterized by the activation of glial cells and the expression of inflammatory mediators [3]. In particular, microglia are responsible for the upregulation of neurotoxic free radicals and various cell surface receptors and inflammatory molecules [46]. Previous studies have shown that Aβ can trigger an immune response that activates microglia to engulf Aβ filaments [2,46,47]. However, excessive microglial activation is detrimental because it can increase the expression of TLR-2 and TLR-4 in microglial cells, thereby leading to the secretion of Aβ [48]. Moreover, overactivated microglia secrete inflammatory cytokines and neurotoxic mediators such as nitric oxide (NO) and ROS, which lead to progressive neuronal damage [47]. Thus, activated microglia contribute to the progression of AD by forming a positive feedback loop that damages neurons and stimulates Aβ [48]. When Aβ accumulates, microglia carrying TLR-4 can detect it and activate the NF-κB pathway through adaptor proteins such as MyD88 [47]. NF-κB promotes the secretion of inflammatory cytokines such as IL-1β and TNF-α. Previous studies have shown that IL-6, IL-1β, and TNF-α were increased in the brain and plasma of AD patients, which suggests that neuroinflammation may contribute to AD [46,49]. Therefore, controlling and managing neuroinflammation may be an important factor that can help prevent or reduce the progression of AD. In this study, EE down-regulated the TLR-4/NF-κB signaling pathway in Aβ-induced neuroinflammation (Figure 6). Phlorotannins extracted from edible brown algae are well known to have powerful anti-inflammatory effects [23]. In a previous study, *E. cava* also decreased lipopolysaccharide (LPS)-induced NF-κB and signal transducer and activator of transcription (STAT)3 activation in mice cerebrum [50]. In addition, *E. cava* inhibited prostaglandin E2, cyclooxygenase (COX)-2, and IL-6 in LPS-stimulated HGF-1 cells [51]. A 7-phloroeckol from *E. cava* inhibited the expressions of p-IκB-α and p-NF-κB in alcohol-induced HepG2/CYP2E cells [45]. These results suggest that phlorotannins-rich EE can contribute to preventing cognitive decline by reducing Aβ-induced inflammation response via the NF-κB pathway.

Several studies have proposed that persistent inflammation in the periphery can induce pro-inflammatory cytokines to cross the BBB, potentially exacerbating cognitive decline in patients with AD [44,46,49]. In addition, Aβ present in the blood vessels can enter the brain through the BBB [7]. In contrast, IDE primarily breaks down and removes Aβ that enters the brain through the BBB. In addition, the BBB detects Aβ molecules with specific transporters and receptors such as low-density lipoprotein receptor-related protein (LRP)1 and p-glycoprotein, and it plays a role in expelling some Aβ molecules from the brain [52]. However, increased BBB permeability makes Aβ clearance difficult, which is observed in early cognitive impairment [28]. The BBB forms a tight junction (TJ) that controls the exchange of substances between the blood and brain tissue by minimizing the gaps between cells [53]. TJ proteins allow for the selective passage of substances, which plays a critical role in maintaining the brain environment and protecting important brain functions [52]. Claudins and occludin play a role in strengthening the connection between cells and sealing the spaces between cells [52]. In particular, claudin-1 and claudin-5 are mainly expressed in the endothelial cells of the BBB [54]. In addition, ZO proteins play a role in anchoring claudins and occludin to the cell membrane, and there are various subunits such as ZO-1, ZO-2, and ZO-3 [53]. Previous studies have suggested that Aβ-mediated oxidative stress and apoptosis may lead to BBB destruction and increase the influx of inflammatory substances, which may affect the onset and progression of neurodegenerative diseases [44,46,54]. Therefore, in this study, we investigated whether EE affects the expression of TJ proteins related to Aβ-induced BBB permeability (Figure 7). As a result, EE increased the expressions of IDE, claudin-1, occludin, and ZO-1, while it decreased the expression of Aβ. Our results indicate that EE enhances the BBB function by activating IDE to degrade Aβ and preserve TJ proteins. Studies on the availability of phlorotannins in brain cells are lacking, and only dieckol is known to be able to penetrate the brain through the BBB [55]. These results show that dieckol-rich EE can directly penetrate into the brain and help improve cognitive function by regulating the concentration of Aβ in the brain.

BDNF is a protein produced primarily by neurons and plays a critical role in the survival, growth, and maintenance of neuronal function [56]. Several studies have demonstrated that BDNF deficiency initiates in the early stages of AD and ultimately results in neurodegeneration, cell death, and loss of cholinergic neurotransmission in the late stages [56,57]. BDNF promotes cell survival by binding to and activating the TrkB cell surface receptor. After binding, BDNF triggers multiple intracellular signaling pathways through TrkB, such as the PI3K/AKT pathway, which plays a crucial role in shaping the development and functionality of the nervous system [58]. PI3K activation phosphorylates AKT, which, in turn, phosphorylates GSK-3β, thereby preventing p-tau accumulation [33,59]. In addition, AKT can phosphorylate CREB and, thus, increase BDNF production [58]. Indeed, brains of AD patients and in vitro neurons treated with Aβ showed decreased p-CREB. Furthermore, it was demonstrated that Aβ induced hippocampal synapse loss and impaired synaptic plasticity, and cognitive deficits were mediated by alterations in BDNF/CREB signaling [56,59]. In this study, it was confirmed that BDNF/AKT/CREB signaling pathways were down-regulating in Aβ-induced mice (Figure 8). In a previous study, a phlorotannin-rich fraction from *Ishige foliacea* brown seaweed increased BDNF and CREB levels in the cerebral cortex and hippocampus of scopolamine-induced mice [32]. Moreover, in our prior study, the water extract of *E. cava* restored the PM_2.5_-induced decrease in BDNF expression [13]. However, studies on the improvement effect of *E. cava* on the BDNF/CREB signaling pathway are still lacking. Our results demonstrate that EE can promote PI3K/AKT activation by TrkB activation, leading to CREB increasing the production of BDNF. The activation of AKT can control the hyperphosphorylation of tau by inactivating GSK-3β and inhibit apoptosis by regulating BCL-2 family proteins [58]. Therefore, we confirmed that the apoptosis and p-tau protein formation inhibitory activities of EE shown in this study (Figure 5) are related to BDNF/AKT/CREB signaling.

In addition, BDNF promotes synaptic strengthening and neuroplasticity in the brain and is essential for various neuronal functions such as learning, memory, and emotional regulation [56]. Synapses in the brain are the connections between neurons, and chemical and electrical signals are transmitted through synapses for the performance of various cognitive functions [60]. The plasticity of synapses plays a central role in learning new information and remembering existing information. ACh is a representative neurotransmitter for chemical signal transmission in neurons, and it is synthesized by combining acetyl-CoA and choline by ChAT in the nerve terminal [2]. Moreover, AChE prevents the imbalanced accumulation of ACh by re-degrading ACh, thereby regulating neurotransmission processes [2]. On the one hand, AChE can bind directly to presenilin-1 (PS-1), accelerating Aβ production and worsening cognitive dysfunction [9]. Aβ can also increase AChE activity in the hippocampus, prefrontal cortex, and amygdala, which may lead to memory impairment [9]. Moreover, Aβ-induced neuroinflammation and oxidative stress may impair learning and memory abilities by interfering with synaptic signaling pathways. SYP is a major synaptic vesicle protein that plays a role in synaptic plasticity by regulating the storage and release of neurotransmitters [61]. PSD-95 is located in postsynaptic nerve terminals and alters synaptic plasticity by regulating the expression levels of synaptic scaffold proteins [61]. Therefore, the expression of synaptic proteins is closely related to the robustness of interneural signaling. In AD, the cholinergic hypothesis was proposed earlier than the amyloid hypothesis, and their interaction has already been extensively demonstrated in many studies [9,14,62]. According to Hidisoglu et al., Aβ_1-42_ peptide injection reduced the expression of pre- (synaptosome associated protein 25 and SYP) and post-synaptic (PSD-95) proteins in the hippocampus of rats [63]. In contrast, dieckol and phlorofucofuroeckol were reported to help improve memory in ethanol-treated mice by inhibiting AChE [22]. Moreover, the inhibitory effects of phlorotannins on AChE and butyrylcholinesterase (BuChE) were revealed in vitro [21]. However, studies about the improvement effects of *E. cava* and phlorotannins on the synaptic functions in animal models are still lacking. The results of this study showed that EE enhanced synaptic plasticity by regulating cholinergic system and synapse proteins of the whole brain, hippocampus, and cerebral cortex in Aβ-induced mice (Figure 9). These findings indicate that dieckol-rich EE can enhance cognitive function through the amelioration of Aβ-induced neurotoxicity by improving synaptic function and structure.

## 5. Conclusions

This study was conducted to further enhance the neuroprotective effect of the water extract of *E. cava* confirmed in our previous study [13] and to create industrial applicability such as functional food. In summary, our results demonstrate that the 70% ethanol extract of *E. cava* (EE) containing various polyphenols (phlorotannins) significantly protects against Aβ-induced cognitive dysfunction in ICR mice. In particular, it was confirmed that the dieckol content is quantitatively superior in the EE compared to the water extract of *E. cava*. Aβ caused oxidative stress by decreasing endogenous antioxidant levels and mitochondrial function in brain tissue. However, EE not only restored this but was also effective in reducing neuroapoptosis and neuroinflammation via the JNK and NF-κB pathway. EE also maintained BBB function by improving TJ protein reduction caused by Aβ toxicity. Furthermore, EE prevented tau hyperphosphorylation and neuroapoptosis through the BDNF/AKT/CREB signaling pathway and increased the expression of synaptic proteins. These protective benefits of EE are assumed to support the upkeep of normal cholinergic systems and ameliorate synaptic plasticity and functionality. In conclusion, this study proposes that EE could be used as an ingredient in functional foods to enhance memory and cognitive function by improving oxidative stress and synaptic functions.

## Figures and Tables

**Table 1:** List of primary and secondary antibodies information used in this study.

Antibody	Catalog	Manufacturer
p-c-Jun N-terminal kinase (p-JNK)	sc-6254	Santa Cruz Biotech (Dallas, TX, USA)
p-Glycogen synthase kinase-3β (p-GSK-3β)	sc-373800	Santa Cruz Biotech
p-Tau	sc-32275	Santa Cruz Biotech
B-cell leukemia/lymphoma (BCL)-2	sc-7382	Santa Cruz Biotech
BCL-2 associated X (BAX)	sc-7480	Santa Cruz Biotech
Caspase-3	sc-56053	Santa Cruz Biotech
Toll-like receptor (TLR)-4	sc-293072	Santa Cruz Biotech
Myeloid differentiation primary response 88 (MyD88)	sc-74532	Santa Cruz Biotech
p-Nuclear factor kappa B (p-NF-κB)	sc-136548	Santa Cruz Biotech
p-NF-κB inhibitor alpha (p-IκB-α)	sc-8404	Santa Cruz Biotech
Interleukin 1β (IL-1β)	sc-515598	Santa Cruz Biotech
Tumor necrosis factor α (TNF-α)	sc-33639	Santa Cruz Biotech
Integrated development environment (IDE)	sc-393887	Santa Cruz Biotech
Aβ	sc-28365	Santa Cruz Biotech
Claudin-1	sc-166338	Santa Cruz Biotech
Occludin	sc-133256	Santa Cruz Biotech
Zonula occludens-1 (ZO-1)	sc-33725	Santa Cruz Biotech
AChE	sc-373901	Santa Cruz Biotech
Synaptophysin (SYP)	sc-17750	Santa Cruz Biotech
Postsynaptic density protein (PSD-95)	sc-32290	Santa Cruz Biotech
Tropomyosin receptor kinase B (TrkB)	sc-377218	Santa Cruz Biotech
Phosphoinositide 3-kinase (PI3K)	sc-1637	Santa Cruz Biotech
p-Protein kinase B (p-AKT)	sc-514032	Santa Cruz Biotech
Choline acetyltransferase (ChAT)	# 27269	Cell Signaling Tech (Danvers, MA, USA)
Brain-derived neurotrophic factor (BDNF)	#47808	Cell Signaling Tech
p-cAMP-response element binding protein (p-CREB)	#9198	Cell Signaling Tech
β-actin	sc-69879	Santa Cruz Biotech
Goat-anti-mouse IgG	AP124P	Sigma-Aldrich Chemical Co.
Goat-anti-rat IgG	#7077	Cell signaling Tech
Goat-anti-rabbit IgG	#7074	Cell signaling Tech

**Table 2:** Identified physiological compounds in 70% ethanol extract of *Ecklonia cava* by UPLC-Q-TOF/MS system.

No.	Retention Time (RT)	Proposed Compound Name	*m*/*z* [M-H]-	Fragments
1	3.24	Triphloroethol/fucophloroethol	373	233, 247, 229, 124, 189
2	4.55	Eckol	371	149, 201, 217, 245, 263
3	4.64	7-Phloroeckol	495	154, 263, 297, 387
4	4.82	6,6′-Bieckol	741	201, 229, 371, 477
5	4.88	6,8′-Bieckol	741	260, 371, 479, 615
6	5.30	Dibenzodioxin-fucodiphloroethol	743	125, 139, 231, 353
7	6.02	Diekcol	741	201, 229, 261, 369, 371, 493, 615
8	6.89	Phlorofucofuroeckol A	601	244, 299, 385, 492, 493
9	6.96	2,7″-Phloroglucinol-6,6′-bieckol (PHB)	973	229, 493, 601, 602, 741, 707, 973

**Table 3:** Content of dieckol in 70% ethanol and water extract of *Ecklonia cava*.

	Dieckol Content
70% ethanol and water extract of *Ecklonia cava* (EE)	90.89 ± 0.20
Water extract of *Ecklonia cava* (WEE)	14.08 ± 0.35

Unit: μg/mg of dried weight. The results are expressed as the mean ± SD (*n* = 3).

## Data Availability

All of the data is contained within the article and the Supplementary Materials.

## Supplementary Materials

The following supporting information can be downloaded at: https://www.mdpi.com/article/10.3390/antiox13080951/s1, Figure S1: UV spectra of 70% ethanol extract of Ecklonia cava (a) and water extract of Ecklonia cava (b) at 254 nm.

## Abbreviations

AD: Alzheimer’s disease; Aβ: amyloid beta; APP: amyloid precursor protein; ROS: reactive oxygen species; MMP: mitochondrial membrane potential; BBB: blood–brain barrier; ACh: acetylcholine; PM_2.5_: particulate matter; *E. cava*: *Ecklonia cava*; EDTA: ethylene-diamine-tetraacetic acid; OPT: o-phthaldialdehyde; EGTA: ethylene glycol-bis (β-aminoethyl ether)-N,N,N′,N′-tetraacetic acid; HEPES: hydroxyethyl piperazine ethane sulfonic acid; DCF-DA: dichloro-dihydro-fluorescein diacetate; JC-1: 1,1′,3,3′-tetraethyl-5,5′,6,6′-tetrachloroimidacarbocyanine iodide; DTNB: 5,5′-dithiobis-2-nitrobenzoic acid; EE: 70% ethanol extract of *E. cava*; WEE: water extract of *E. cava*; DAD-HPLC: high-performance liquid chromatography with diode-array detection; ECL: enhanced chemiluminescence; p-JNK: p-Jun N-terminal kinase; p-GSK-3β: p-glycogen synthase kinase; BCL-2: B-cell leukemia/lymphoma-2; BAX: BCL-2 associated X; TLR-4: toll-like receptor-4; MyD88: myeloid differentiation primary response 88; p-NF-κB: p-nuclear factor-kappa B; p-IκB-α: p-NF-κB inhibitor alpha; IL-1β: interleukin 1 beta; TNF-α: tumor necrosis factor alpha; IDE: integrated development environment; ZO-1: zonula occludens-1; SYP: synaptophysin; PSD-95: postsynaptic density protein-95; TrkB: tropomyosin receptor kinase B; PI3K: phosphoinositide 3-kinase; AKT: protein kinase B; ChAT: choline acetyltransferase; BDNF: brain-derived neurotrophic factor; CREB: cAMP-response element binding protein; PHB: 2,7″-Phloroglucinol-6,6′-bieckol; UPLC-Q-TOF-MS: ultra-performance liquid chromatography-quadrupole time-of-flight mass spectrometry; RT: retention time; NC: normal control; PA: passive avoidance; MWM: Morris water maze; MDA: malondialdehyde; TBARS: thiobarbituric acid reactive substances; TBA: thiobarbituric acid; GSH: glutathione; SOD: superoxide dismutase; WST: water-soluble tetrazolium; AChE: acetylcholinesterase; ECL: enhanced chemiluminescence; ERK: extracellular signal-regulated kinase; NADPH: nicotinamide adenine dinucleotide phosphate hydrogen; Nrf2: nuclear factor erythroid 2-related factor 2; HO-1: heme oxygenase-1; HFD: high-fat diet; TMT: trimethyltin; Gpx: glutathione peroxidase; Apaf-1: apoptotic protease activating factor 1; NFTs: neurofibrillary tangles; MAPK: microtubule-affinity regulating kinase; STAT: signal transducer and activator of transcription; LPS: lipopolysaccharide; COX-2: cyclooxygenase; LRP: low-density lipoprotein receptor-related protein; TJ: tight junction; PS-1: presenilin-1; BuChE: butyrylcholinesterase.
