# Supplementary material for: Ecklonia cava Ameliorates Cognitive Impairment on Amyloid β-Induced Neurotoxicity by Modulating Oxidative Stress and Synaptic Function in Institute of Cancer Research (ICR) Mice
Source: Antioxidants (Basel). 2024 Aug 6;13(8):951. doi: 10.3390/antiox13080951 (PMC11352165; doi:10.3390/antiox13080951)
Supplement: Supplementary file 1 [file antioxidants-13-00951-s001.zip › antioxidants-3112919-supplementary.pdf]

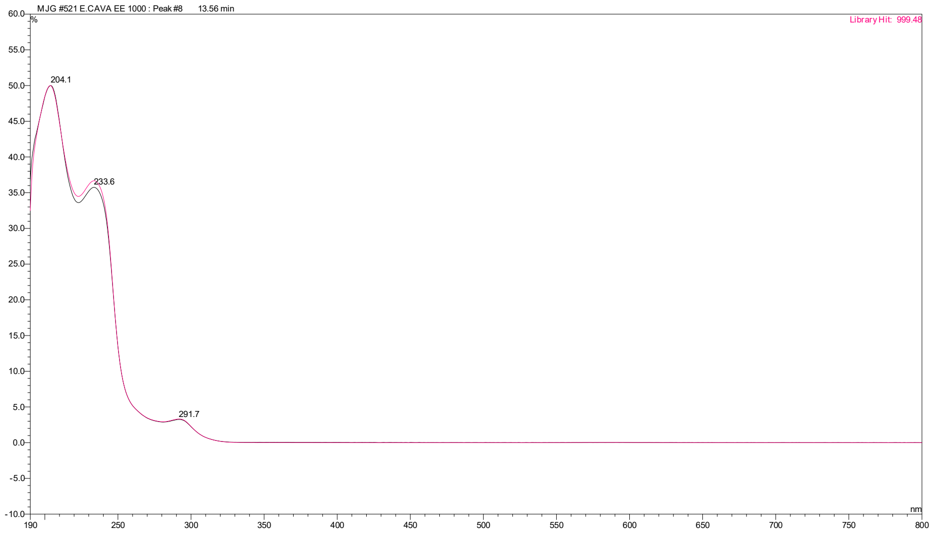

(**a**)

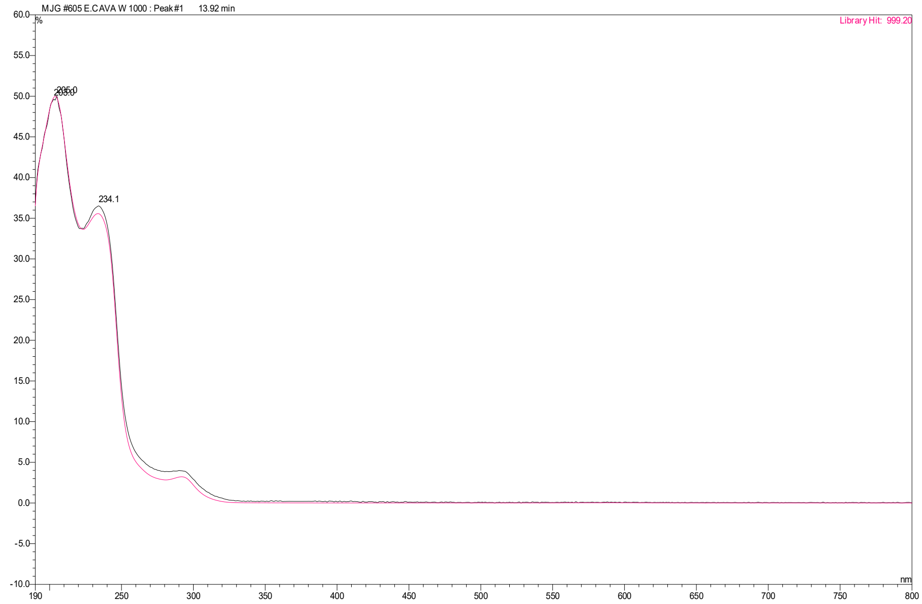

(**b**)

Figure S1. UV spectra of 70% ethanol extract of *Ecklonia cava* (**a**) and water extract of *Ecklonia cava* (**b**) at 254 nm.
